# Optimal strategies to improve uptake of and adherence to HIV prevention among young people at risk for HIV acquisition in the USA (ATN 149): a randomised, controlled, factorial trial

**DOI:** 10.1016/S2589-7500(23)00252-2

**Published:** 2024-03

**Authors:** Dallas Swendeman, Mary Jane Rotheram-Borus, Elizabeth Mayfield Arnold, Maria Isabel Fernández, Walter Scott Comulada, Sung-Jae Lee, Manuel A Ocasio, Kelsey Ishimoto, William Gertsch, Naihua Duan, Cathy J Reback, Debra A Murphy, Katherine A Lewis

**Affiliations:** Semel Institute for Neuroscience and Human Behavior, University of California, Los Angeles, CA, USA; Semel Institute for Neuroscience and Human Behavior, University of California, Los Angeles, CA, USA; Department of Psychiatry, College of Medicine, University of Kentucky, Lexington, KY, USA; College of Osteopathic Medicine, Nova Southeastern University, Fort Lauderdale, FL, USA; Semel Institute for Neuroscience and Human Behavior, University of California, Los Angeles, CA, USA; Semel Institute for Neuroscience and Human Behavior, University of California, Los Angeles, CA, USA; Department of Pediatrics, Tulane University School of Medicine, New Orleans, LA, USA; Semel Institute for Neuroscience and Human Behavior, University of California, Los Angeles, CA, USA; Semel Institute for Neuroscience and Human Behavior, University of California, Los Angeles, CA, USA; Division of Mental Health Data Science, Department of Psychiatry, Columbia University, New York, NY, USA; Friends Research Institute, Los Angeles, CA, USA; Semel Institute for Neuroscience and Human Behavior, University of California, Los Angeles, CA, USA; Semel Institute for Neuroscience and Human Behavior, University of California, Los Angeles, CA, USA

## Abstract

**Background:**

Pre-exposure prophylaxis (PrEP), condom use, post-exposure prophylaxis (PEP), and sexual partner reduction help to prevent HIV acquisition but have low uptake among young people. We aimed to assess the efficacy of automated text messaging and monitoring, online peer support, and strengths-based telehealth coaching to improve uptake of and adherence to PrEP, condom use, and PEP among adolescents aged 12–24 years at risk of HIV acquisition in Los Angeles, CA, USA, and New Orleans, LA, USA.

**Methods:**

We conducted a four-arm randomised controlled factorial trial, assessing interventions designed to support uptake and adherence of HIV prevention options (ie, PrEP, PEP, condom use, and sexual partner reduction). We recruited young people aged 12–24 years who were at risk of HIV acquisition from 13 community-based organisations, adolescent medicine clinics, and organisations serving people who are unstably housed, people who were previously incarcerated, and other vulnerable young people, and through dating apps, peer referrals, and social venues and events in Los Angeles, CA, USA, and New Orleans, LA, USA. Young people who tested seronegative and reported being gay, bisexual, or other men who have sex with men, transgender men or women, or gender diverse (eg. non-binary or genderqueer) were eligible for inclusion. Participants were randomly assigned to one of four intervention groups in a factorial design: automated text messaging and monitoring (AMMI) only, AMMI plus peer support via private social media, AMMI plus strengths-based telehealth coaching by near-peer paraprofessionals, or AMMI plus peer support and coaching. Assignment was further stratified by race or ethnicity and sexual orientation within each interviewer’s group of participants. Participants were masked to intervention assignment until after baseline interviews when offered their randomly assigned intervention, and interviewers were masked throughout the study. Interventions were available throughout the 24-month follow-up period, and participants completed baseline and follow-up assessments, including rapid diagnostic tests for sexually transmitted infections, HIV, and substance use, at 4-month intervals over 24 months. The primary outcomes were uptake and adherence to HIV prevention options over 24 months, measured by self-reported PrEP use and adherence, consistent condom use with all partners, PEP prescription and adherence, and number of sexual partners in participants with at least one follow-up. We used Bayesian generalised linear modelling to assess changes in outcomes over time comparing the four study groups. This study is registered with ClinicalTrials.gov (NCT03134833) and is completed.

**Findings:**

We screened 2314 adolescents beginning May 1, 2017, to enrol 1037 participants (45%) aged 16–24 years between May 6, 2017, and Aug 30, 2019, of whom 895 (86%) had follow-up assessments and were included in the analytical sample (313 assigned to AMMI only, 205 assigned to AMMI plus peer support, 196 assigned to AMMI plus coaching, and 181 assigned to AMMI plus peer support and coaching). Follow-up was completed on Nov 8, 2021. Participants were diverse in race and ethnicity (362 [40%] Black or African American, 257 [29%] Latinx or Hispanic, 184 [21%] White, and 53 [6%] Asian or Pacific Islander) and other sociodemographic factors. At baseline, 591 (66%) participants reported anal sex without a condom in the past 12 months. PrEP use matched that in young people nationally, with 101 (11%) participants reporting current PrEP use at baseline, increasing at 4 months to 132 (15%) and continuing to increase in the AMMI plus peer support and coaching group (odds ratio 2·31, 95% CI 1·28–4·14 *vs* AMMI control). There was no evidence for intervention effect on condom use, PEP use (ie, prescription or adherence), PrEP adherence, or sexual partner numbers. No unanticipated or study-related adverse events occurred.

**Interpretation:**

Results are consistent with hypothesised synergistic intervention effects of evidence-based functions of informational, motivational, and reminder messaging; peer support for HIV prevention; and strengths-based, goal-focused, and problem-solving telehealth coaching delivered by near-peer paraprofessionals. These core functions could be flexibly scaled via combinations of technology platforms and front-line or telehealth HIV prevention workers.

## Introduction

Despite decades of research developing evidence-based interventions to prevent HIV among adolescents (ie, aged 12–24 years), prevalence and incidence rates are still unacceptably high.^1^ More than 80% of young people living with HIV identify as sexual or gender minorities, primarily gay, bisexual, and other men who have sex with men (GBMSM) and transgender women, with increased incidence and prevalence among Black or African American and Hispanic or Latinx young people.^1^ Young people with mental ill health, substance misuse issues, or housing insecurity are at increased risk for acquiring HIV, reflecting the effect of syndemic factors and competing hierarchies of needs.^2^ Young people who identify as sexual or gender minorities are also at increased risk for homelessness due to familial ejection with disclosure of sexual or gender minority identities.^3^ Stigma and discrimination associated with intersecting minority sexual, gender, racial, and ethnic status also disrupt employment and economic security, which might contribute to engagement in sex work.^3^ These often-cited barriers to HIV prevention among populations at risk of HIV lead to critiques of narrowly focused interventions that do not address adolescents’ competing needs and increasingly digital lives.^4^

Condom use is crucial for preventing sexually transmitted infections (STIs) but has failed to stem the HIV epidemic, thus, HIV pre-exposure prophylaxis (PrEP) has become a crucial component of comprehensive HIV prevention with potential to nearly eliminate HIV acquisition.^5^ However, PrEP uptake and adherence is low among young people, particularly Black and Latinx young people. The US Centers for Disease Control and Prevention (CDC) estimates that in 2019, when enrolment for this study ended, only 275 794 (22·7%) of 1 216 210 people in the USA who would benefit from PrEP used it; rates were lowest among young people aged 16–24 years (36 969 [15·0%] of 246 290), female adolescents (21 095 [9·3%] of 227 010), and Latinx (45 392 [14·5%] of 312 820) and Black or African American (37 499 [8·0%] of 468 540) individuals.^1^

PrEP uptake and adherence interventions are still new and incorporate many efficacious HIV prevention strategies, such as coaching, peer support, and automated texting to encourage uptake and adherence.^6^ To date, the US CDC have identified two evidence-based interventions and two evidence-informed interventions (ie, interventions with promising evidence) that improved PrEP uptake for sexual and gender minority populations in the USA that included young people, but the interventions were brief, with duration of effects or follow-up of 3 months or less.^7–10^ The two evidence-based interventions also included adult GBMSM, with one intervention using text and video messages delivered via smartphones^8^ and the other intervention using brief motivational interview-based counselling.^9^ The two evidence-informed interventions targeted adolescent GBMSM, with one intervention using tailored counselling with PrEP linkage support and one follow-up session among Black individuals aged 16–25 years who have sex with men^7^ and the other intervention using a four-module, self-directed, web-based sexual education curriculum among men aged 13–18 years who have sex with men.^10^

It is crucial that the evidence base for HIV prevention interventions developed during the past 30 years is reviewed to select the strongest components to prevent HIV acquisition among young people. Additionally, most interventions are not scaled up, not delivered with fidelity to manuals, or not sustained in real-world practice due partly to lack of adaptability or flexibility for front-line organisations, staff, or diverse client needs.^4^ Disruptive innovations in prevention interventions are warranted that use simpler, flexible, and more adaptable modes (eg, non-manualised, paraprofessional, automated technology, social media, and self-directed strategies) rather than highly specialised and scripted approaches.^4^ Knowledge generated on components of evidence-based interventions should be used to improve the flexibility and scalability of interventions for real-world implementation.^4^ For example, a meta-analysis of interventions supporting HIV care outcomes identified that patient navigation, appointment help or alerts, and psychosocial support were effective in improving re-engagement and retention in care and viral suppression.^11^ This evidence is not novel, but points to well established components of competent health care.

Mobile phone technology interventions hold promise for high reach and decreased costs relative to traditional counselling modalities.^4^ More than 95% of US teenagers have a mobile phone, and texting or direct messaging on apps is the preferred communication method for many young people.^12^ Automated text messaging has shown potential for improving HIV and STI protective behaviours, such as condom use,^13,14^ but mixed evidence exists for PrEP uptake or adherence.^15^

Peer support has long been identified as efficacious for reducing HIV risk.^16^ Adolescents are especially influenced by peer behaviours and norms, both for risky and protective behaviours.^17^ HIV and STI peer education has had positive effects on knowledge, attitudes, normative beliefs, and self-efficacy, but equivocal findings on effects have been reported for behaviours.^18^ Peer support has been delivered via online social networks (eg, Facebook) to effectively increase condom use and HIV testing but, to date, these platforms have not been shown to increase PrEP use.^19^

Many counselling-based interventions (ie, coaching)^4,20^ have reduced HIV risk using cognitive–behavioural strategies.^20^ For example, cognitive counselling implemented by paraprofessional HIV-test counsellors reduced unprotected anal sex among GBMSM, with stronger and more immediate effects than usual client-centred risk-reduction counselling.^21^ Some coaching interventions also address environmental factors that indirectly affect HIV risk, including housing, medical care, employment, and sex work, resulting in decreased condomless sex.^22^ Strengths-based coaching and case management has also gained recognition for application to prevention interventions by emphasising strengths and promoting resilience within participants instead of having a narrow focus on risks and deficits.^23^

Our study was designed to assess HIV prevention interventions on the basis of core evidence-based intervention functions delivered by readily available and adaptable platforms: automated text messaging, mobile web-app peer support, and non-manualised telehealth coaching by paraprofessionals. We hypothesised that the combined intervention would have synergistic rather than additive effects.

## Methods

### Study design and participants

We conducted a four-arm randomised controlled factorial trial in the USA, assigning participants at the individual level to one of four interventions. We recruited young people aged 12–24 years who were at risk for HIV acquisition in Los Angeles, CA, USA, and New Orleans, LA, USA, from 13 community-based organisations serving young people who identify as a sexual or gender minority, adolescent medicine clinics, and organisations serving people who are unstably housed, people who were previously incarcerated, and other vulnerable young people. Participants were also recruited through dating apps (eg, Grindr, Jack’d, and Scruff),^24^ peer referrals, and social venues (eg, bars, clubs, and community events). Young people were considered to be at risk of HIV acquisition and eligible for inclusion if they tested seronegative for HIV and reported being GBMSM, transgender (male or female), or gender diverse (eg, non-binary or genderqueer). Current behavioural risk for HIV acquisition was not required to be inclusive of adolescents who had not yet initiated or had intermittent behavioural risks to anticipate developmental transitions (eg, experimentation and new relationships) and potential under-reporting of sensitive behaviours at screening. Exclusion criteria were inability to provide voluntary written informed consent or understand study procedures due to intoxication or cognitive difficulties.

Interviewers verbally administered eligibility screening and conducted rapid HIV testing. Screening required verbal consent for potential participants aged 15–24 years and written assent for potential participants aged 12–14 years, per the University of California, Los Angeles institutional review board. Signed informed consent or assent was obtained for study enrolment. The University of California, Los Angeles institutional review board waived parental permission for minor enrolment (ie, under the age of 18 years) because this study was not considered greater than minimal risk and obtaining parental permission was not a reasonable requirement according to the following criteria: informing the parent of the participant’s study eligibility or participation might expose the child to risk and obtaining parental permission would violate the child’s confidentiality regarding issues such as reproductive health, mental health, drug and alcohol use, and HIV status. Child assent was obtained by interviewers, who were not involved in their medical or psychological care.

All procedures were approved by the University of California, Los Angeles institutional review board (IRB number 16–001674-AM-00005) and the Adolescent Medicine Trials Network (ATN) Study Monitoring Committee. The protocol has been previously published.^20^

### Randomisation and masking

Interviewers enrolled participants, who were then randomly assigned by the lead statistician to one of four study groups by use of computer-generated random-number tables for each interviewer group, further stratified to ensure diversity of race or ethnicity and sexual orientation in each group. Randomisation was administered automatically through the mobile-web app for study screening, assessment, case management, and intervention (ATN CARES CommCare, Dimagi, Cambridge, MA, USA). Participants were masked to intervention assignment until after baseline interviews, when they received text messages notifying them of intervention assignment and follow-up by study coaches for peer support and coaching interventions. Interviewers verbally administered the baseline interviews and were masked to intervention assignment throughout the study. We did not assess success of masking. The original block randomisation assigned 60·0% of participants to the control group (ie, automated text messaging and monitoring [AMMI] only), 13·3% to AMMI plus peer support, 13·3% to AMMI plus coaching, and 13·3% to AMMI plus peer support and coaching. This technique was designed to achieve power for the intervention groups with peer support or coaching, or both, and create a larger sample for the secondary aim to identify acute or untreated HIV infections for a parallel study protocol to monitor HIV reservoirs over time (ATN 147).^25^ A funder-initiated protocol change executed on Jan 22, 2019, to focus exclusively on GBMSM and gender-diverse young people (excluding cisgender heterosexual men and cisgender women after 12-month follow-up) to meet funding constraints resulted in updated power calculations and change in randomisation scheme to 30·0% for each of AMMI plus peer support, AMMI plus coaching, and AMMI plus peer support and coaching and 10·0% for AMMI only.

### Procedures

Study procedures have been previously described elsewhere.^20^ Briefly, participants completed baseline and follow-up assessments, including rapid diagnostic tests for STIs, HIV, and substance use, at 4-month intervals over 24 months. Assessments were administered verbally by near-peer interviewers. Participants received US$50 cash incentives for each assessment. Following each assessment, interviewers provided service referrals, including for PrEP. All interventions were available for the entire 24-month follow-up period. Race, and ethnicity (ie, Latinx or Hispanic), were assessed in two separate questions via self-report from a list of response options, where more than one response was allowed and an open-ended option was included for participants who preferred to provide an additional response.

Anticipated risks from study participation were emotional or psychological distress in responding to study questions, receiving positive HIV or sexually transmitted infection test results, or physical discomfort from HIV and sexually transmitted infection testing procedures. Anticipated adverse events unrelated to the study were suicidal ideations or attempts. Adverse events were reported within 24 h to the sponsor’s project scientist for confirmation of unanticipated or study-related events. Adverse events were also reported to the study monitoring committee biannually and the institutional review board annually.

AMMI was provided to all participants due to ethical considerations associated with providing some intervention with evidence for efficacy for HIV prevention. AMMI consisted of unidirectional (ie, non-interactive) daily informational, motivational, and reminder text messages and a weekly self-monitoring survey. We adapted existing message libraries,^14^ including from PrEPTech, with input from a youth advisory board, resulting in libraries of more than 400 theory-based and evidence-based messages. Up to five messages were sent daily in five content streams on physical health and health care, mental health and wellness, sexual health, substance use, and medication reminders (if applicable). Messages on sexual health and substance use were sent only on Thursdays to Saturdays, per feedback from the youth advisory board. Within each domain, messages were randomly repeated to create lists of 730 messages for 24 months of daily messages and 312 sexual health and substance use messages for 3 days per week for 24 months. Participants could opt out of individual message streams or stop all messages. The number of PrEP-specific messages was intentionally low due to feedback from the youth advisory board suggesting that participants might perceive the project to be pushing PrEP, because PrEP was not approved by the US Food and Drug Administration for adolescents younger than 18 years until study midpoint, and to minimise confounding of this active control condition. Notably, only 27 of 312 sexual health messages sent over 24 months were related to PrEP. On Oct, 9, 2020, messages were updated by youth advisory boards in New Orleans, LA, USA, and Los Angeles, CA, USA, and coaching staff. During this process, each message was reviewed and noted to retain, edit with suggested language, or remove. Website links embedded as tiny URLs within text messages were also checked and updated if needed. Text messaging costs about $0·01 per message, with around four messages per day depending on medication adherence messages, opt-outs, and days of the week, for about $15 per year per participant.

Weekly self-monitoring check-in surveys assessed seven domains for the past 7 days: presence of possible acute HIV infection symptoms; presence of STI symptoms; and number of days of feeling sad or depressed, having condomless sex, using alcohol or drugs, not having a place to sleep or enough food, and missing taking medications. Surveys were sent via text message or web survey link in an email. Interviewers attempted HIV or STI symptom follow-up within 2 weeks. Participants received $1 for completing each survey.

In the groups assigned to receive AMMI plus peer support or AMMI plus peer support and coaching, participants were invited by coaches via text message, telephone call, and email invitations to create an account and anonymous user profile and participate in reading and posting comments in a private online discussion board on Muut, an open-source platform similar to Discord. An animated video created by the study team instructed participants in creating a personalised anonymous user profile with avatars and images. Study coaches checked in on discussions at least twice daily, typically spending no more than 1 h moderating and providing evidence-based responses and links to information and referrals. Private messaging functions were disabled due to institutional review board concerns around non-monitored communication between participants. Muut licence fees were $5000 per year for several online discussion boards across ATN CARES protocols. Young people were incentivised with $10 for posting three messages per week for up to 16 weeks.

For strengths-based telehealth coaching, paraprofessional near-peer coaches (ie, similar in age, race or ethnicity, and sexual and gender minority status) supported young people via telephone calls, text messaging, social media direct messaging on participants’ preferred platforms, in person, or via a combination of approaches. Coaches with previous experience as front-line HIV or STI prevention workers (eg, PrEP navigators and health or peer educators) were hired and trained. Salaries ranged from approximately $35 000 to $55 000 annually for a full-time position (ie, approximately $50 000–75 000 including benefits costs) depending on year and study location. Salaries were similar to those of front-line HIV prevention workers. Coach training occurred over 4 weeks in conjunction with institutional review board and staff training and contacting participants to register for peer support. Coaches were certified via role-play demonstrations with a PhD-level clinical psychologist and social worker (EMA). Of 12 coaches hired and trained, only one was not certified. Group and one-on-one supervision of coaching sessions with training boosters were conducted in person and by Zoom (Zoom Video Communications, San Jose, CA, USA), weekly initially and then twice monthly. There were six core elements of coaching. First, the initial strengths assessment conducted in person or remotely via semi-structured interview identifying young people’s perceptions of strengths and challenges in daily living (eg, housing, work, and education), social relationships (eg, family, friends, and partners), physical health, health care, mental health, and sexual health and substance use. The second element was navigation, linkage, and referral to indicated services (eg, housing, employment, insurance, and PrEP) with the goal for each young person being follow-through on scheduling and attending appointments. Coaches ideally called service contacts (eg, PrEP navigators, social workers, and case managers) with participants on three-way calls for referral linkages, including to PrEP programmes, rather than simply providing contact information for the participant to initiate follow-up. Parents or caregivers were not directly involved in coach communications. Third was goal setting, with participants setting up to three goals, with long-term goals narrowed to shorter-term specific, measurable, achievable, relevant, and time-bound goals, including at least one goal on HIV prevention (eg, sexual safety plans, such as use of condoms, PrEP, or post-exposure prophylaxis (PEP); partner strategies; and adherence) or substance use. Other goals focused on young people’s priorities of housing, career (eg, education and employment), health care (eg, insurance, having a regular provider, and gender-affirming care), healthy behaviours (eg, diet and exercise), and relationships. Fourth was problem solving on what worked to achieve goals and what did not work at follow-up coaching sessions, decisional balancing, and setting new short-term goals to achieve long-term goals. Fifth was cognitive and behavioural skills training based on 17 common evidence-based intervention practice elements across prevention interventions for young people applied to topic areas addressed by coaches (appendix p 1).The sixth and final element was follow-up sessions. Frequency and duration were based on participants’ preferences and needs and could vary over time on the basis of developmental transitions or crises. Generally, the aim for coaching was to have weekly 30-min sessions during the first 2 months, then monthly check-ins of 5–20 min, for about 10 h of intervention. Coaches could carry caseloads of approximately 60 participants each, with about a third of participants actively engaged weekly or monthly, a third who were responsive but not engaged, and a third who were non-responsive. Participants who were not engaged or were non-responsive were consistently recontacted by different coaches until study completion or withdrawal.

### Outcomes

Primary outcomes assessed at each timepoint in all participants over 24 months of follow-up and analysed in participants with at least one follow-up visit (ie, the analytical sample) were uptake and adherence to prevention options in the HIV prevention continuum,^26^ operationalised as self-reports of PrEP use (currently) and adherence (6-point Likert scale, reporting none to all doses in past 30 days); consistent condom use with all partners (ie, 100%); PEP prescription (in past 4 months) and adherence (all doses, reported as yes or no); and number of sexual partners. Study procedures providing HIV and STI testing at each study visit confounded HIV and STI status as outcome measures and so were not included as primary outcomes.

Other domains analysed in participants who had at least one follow-up (ie, the analytical sample) included lifetime and recent (in past 4 months) self-reports of substance use and treatment, injection drug use and syringe sharing, sex exchange (ie, formal sex work or informal sex exchange for money, food, goods, or a place to stay), any STIs, housing instability, mental health hospital admissions and symptoms, incarceration or probation, and PrEP knowledge and barriers. We assessed health-care-related factors via self-reports of insurance enrolment, having a regular health-care provider (ie, doctor or clinic), and number of times in past 4 months that participants received care at a doctor’s office or clinic, received care at an emergency room or urgent care, and participated in HIV prevention programmes or events. However, reporting on these factors is outside the scope of this Article and will be reported in a future publication.

### Statistical analysis

We calculated the sample size needed to detect meaningful changes in binary outcome measures (eg, consistent condom use and PrEP use) between two intervention groups over time with 80% power. Our target sample size sufficed to detect 10% or larger increases in one group relative to another over the follow-up period for base rates near zero. Calculations indicated needing 160 young people who identified as a sexual or gender minority per group, but we aimed for more than 200 to buffer against attrition. First, we simulated datasets with repeated observations for each participant generated from a binomial distribution. We generated outcome probabilities from random-effects logistic models parameterised to reflect assessment schedules (ie, seven per participant), assuming no baseline outcome differences and linear slopes across intervention conditions. We used random effects to simulate correlations between repeated participant observations over time. We varied intervention condition sample sizes, regression coefficient values, and random-effect variances to reflect reasonable outcome predicted probabilities and simulated 1000 datasets for each set of parameters. Second, we fitted generalised estimating equation (GEE) models to each simulated dataset. Finally, we estimated power as the ratio of the number of GEE models yielding a significant intervention effect (ie, intervention by time interaction with p<0·05) divided by 1000.

Sociodemographic characteristics, social determinants of health, and behavioural characteristics were compared across groups using χ^2^ tests and one-way ANOVA. Intention-to-treat logistic and linear regression analyses examined effects of intervention conditions on primary outcomes. Models included covariates for intervention group, time from baseline, and intervention by time interactions. We fitted generalised linear mixed-effects models (GLMMs) and GEE models for binary outcomes over time and evaluated the potential effects of missing data, assumed outcome trajectories, and confounders to ensure robustness of results. GLMMs incorporated random effects for each participant and GEE models incorporated an autoregressive (AR[1]) correlation structure to account for correlations between repeated participant observations over time. GLMMs offer two key benefits over GEE: improved efficiency estimating coefficients if distributional assumptions hold, and adjustment for missing outcome observations if missing data mechanisms depended on other observed outcome and covariate observations; GEE adjusts missing outcome observations only on the basis of covariates. GEE models made fewer assumptions than GLMMs, which reduced algorithm complexity and computational burden. Complexity was relevant because some proportions were small (ie, PrEP and PEP use), requiring Bayesian GLMMs instead of maximum likelihood algorithms.

Although GLMMs provided more comprehensive adjustment for missing data than GEE models, both were subject to biased estimation if missing data mechanisms depended on unobserved variables. We examined potential effects of attrition in several ways. First, we fitted GLMMs and GEE models to the analytical sample (ie, participants who had potential intervention exposure and one or more follow-up assessments) and conducted sensitivity analyses also including participants who completed only baseline assessments. Second, we conducted a χ^2^ test for independence between follow-up visit numbers and intervention groups. Third, we compared participants with second-year follow-ups versus participants with only first-year follow-ups and controlled for factors in adjusted models for which differences had been identified.

We tested goodness of fit for GLMMs with linear, quadratic, and cubic time trends, selecting models with best fit on the basis of the widely applicable information criterion. Primary analysis included only covariates for time and intervention effects. This method assumed that randomised intervention assignment addressed potential imbalances in participant character istics across groups. As a safeguard, we also ran GLMMs and GEE models with additional adjustment for baseline covariates that differed significantly between intervention groups (p<0·05), might correlate with outcome variables on the basis of scientific literature (eg, associations of PrEP uptake with race or ethnicity and gender identity),^27,28^ or were associated with lower retention in the χ^2^ tests for independence between study groups, and stratification variables (ie, race or ethnicity and sexual orientation). Analyses also controlled for enrolment date, because participants were more likely to be randomly assigned to AMMI only earlier in recruitment due to the protocol change described earlier, and COVID-19 onset in March, 2020, which might have affected retention and PrEP indications and availability.

We present results for GLMMs fitted to the analytical sample. We favoured GLMMs because they provided additional missing data adjustment. We present results with and without covariates with a focus on those without covariates given the focus on intervention effects.

The study monitoring committee reviewed data and study progress every 6 months.

This study is registered with ClinicalTrials.gov, NCT03134833.

### Role of the funding source

The funder of the study had no role in study design, data collection, data analysis, data interpretation, or writing of the report.

## Results

**Figure 1:**
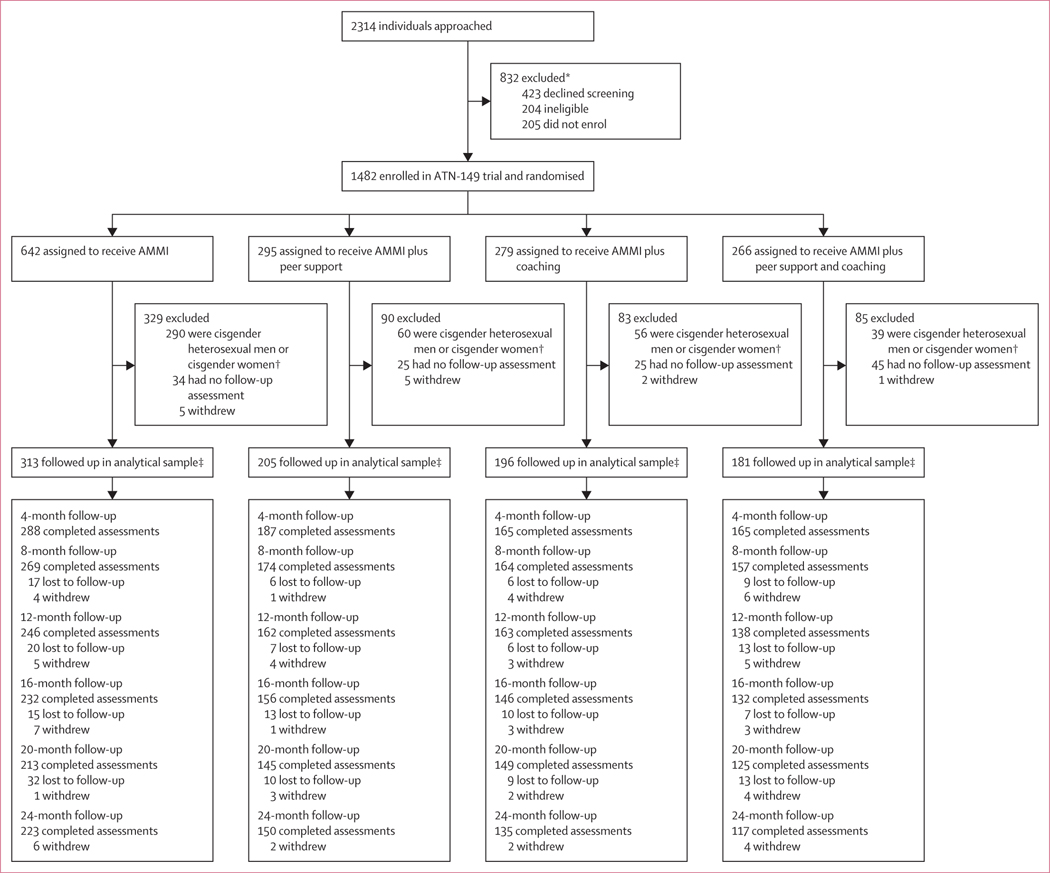
Trial profile. Losses to follow-up indicate that the participant was not seen at that visit or at any subsequent visits. Withdrawals and loss to follow-up indicates a change in the denominator of total participants remaining in the study. Besides the loss to follow-up, there are missing assessments at each timepoint, which indicate people who did not complete a given assessment, but were not entirely lost to follow-up (ie, they later returned and completed an assessment). AMMI=automated text messaging and monitoring. *Including 250 participants with HIV who were enrolled into ATN-147 and ATN-148. †Study terminated at 12 months per sponsor. ‡The analytical sample includes young people who completed at least one follow-up assessment. This group excludes participants who were lost to follow-up, withdrew, or were withdrawn before the 4-month follow-up.

Recruitment occurred from May 1, 2017, to Aug 30, 2019, with the first enrolment completed on May 6, 2017. Follow-ups were completed on Nov 8, 2021. A consecutive series of 2314 young people were approached by interviewers and asked for verbal assent to screen for eligibility, with 423 (18%) declining screening. Of the 1482 people who were enrolled and randomly assigned to an intervention, 445 (30%) cisgender heterosexual male participants and cisgender female participants were subsequently excluded due to the protocol change. The analytical sample was comprised of 895 (86%) of the remaining 1037 GBMSM, transgender, and gender-diverse participants who completed one or more follow-up assessments (figure 1). Most participants were assigned male sex at birth, and reported cisgender identity (table 1). More than half of participants identified as gay and around a quarter identified as bisexual. Black or African American was the most common race or ethnicity reported (including 64 [7%] participants who also reported other races or ethnicities). More than a third of participants had been homeless in their lifetime, and around a sixth had been incarcerated.

605 (75%) of 805 participants reported receiving the text messages at the first 4-month follow-up. In the AMMI plus peer support group, 57 (28%) of 205 individuals participated in peer support activities, with a mean of 31 posts (SD 48; median of 5, IQR 2–45; range 1–203). Of the individuals who participated, 17 (30%) made only one or two posts. In the AMMI plus peer support and coaching group, 49 (27%) of 181 individuals participated, with a mean of 32 posts (SD 55; median of 9, IQR 3–44; range 1–333), of whom 14 (29%) participants made only one or two posts.

In the AMMI plus coaching group, 109 (56%) of 196 participants completed one or more coaching sessions (mean of 13 [SD 15]; median of 8, IQR 3–18; range 1–70); 15 (8%) participants had only one session. In the AMMI plus peer support and coaching group, 92 (51%) of 181 participants had one or more coaching sessions (mean of 9 [SD 10]; median of 4, IQR 2–13; range 1–45); 15 (8%) participants had only one session.

Imbalances between intervention groups were evident for gender identity (χ^2^=9·024, df=3; p=0·029), lifetime homelessness (χ^2^=19·955, df=3; p=0·0002), incarceration (χ^2^=7·955, df=3; p=0·047), popper (ie, nitrite inhalants) use (χ^2^=7·816, df=3; p=0·049), sex exchange (χ^2^=7·923, df=3; p=0·048), and use of support services in the past 4 months (χ^2^=26·835, df=3; p<0·0001). The χ^2^ test for independence showed no significant association between number of follow-up visits and intervention group (χ^2^=22·169, df=15, p=0·10; appendix pp 3–4). When comparing participants with second-year follow-ups versus participants with only first-year follow-ups, participants with only first-year follow-ups had a significantly lower level of education, less use of PrEP, and less use of support services (appendix pp 5–7). The number of participants with hospital admissions for mental health, who had ever been homeless, who had ever been incarcerated, and who had used cannabis in the past 4 months was significantly higher in participants with only first-year follow-ups (appendix pp 5–7). We controlled for these factors in adjusted models.

805 (90%) of 895 participants in the analytical sample completed the follow-up assessment at 4 months, declining steadily over time to 625 (70%) participants at 24 months (figure 1; appendix p 4).

**Figure 2:**
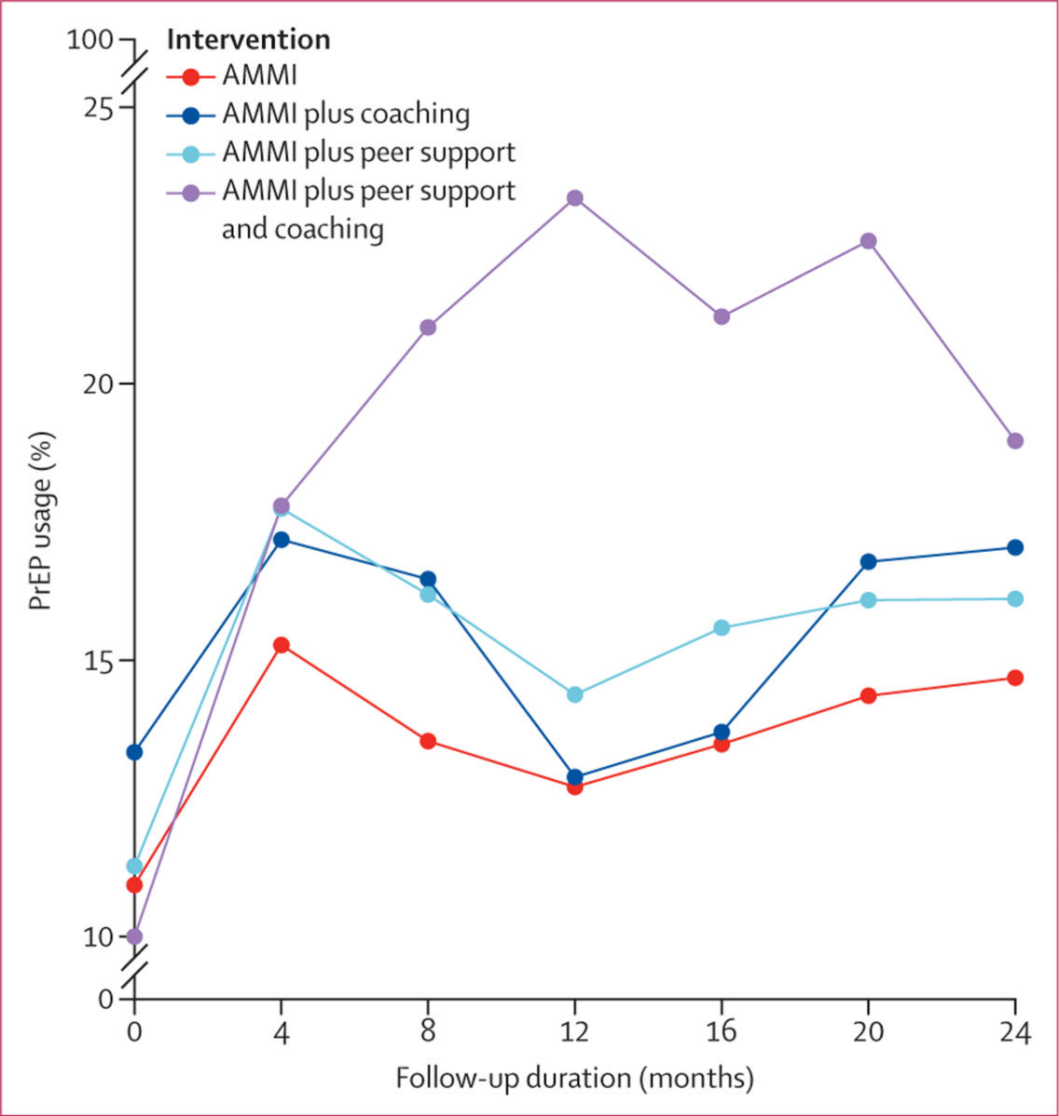
PrEP usage proportions over time by intervention group AMMI=automated text messaging and monitoring. PrEP=pre-exposure prophylaxis.

**Figure 3:**
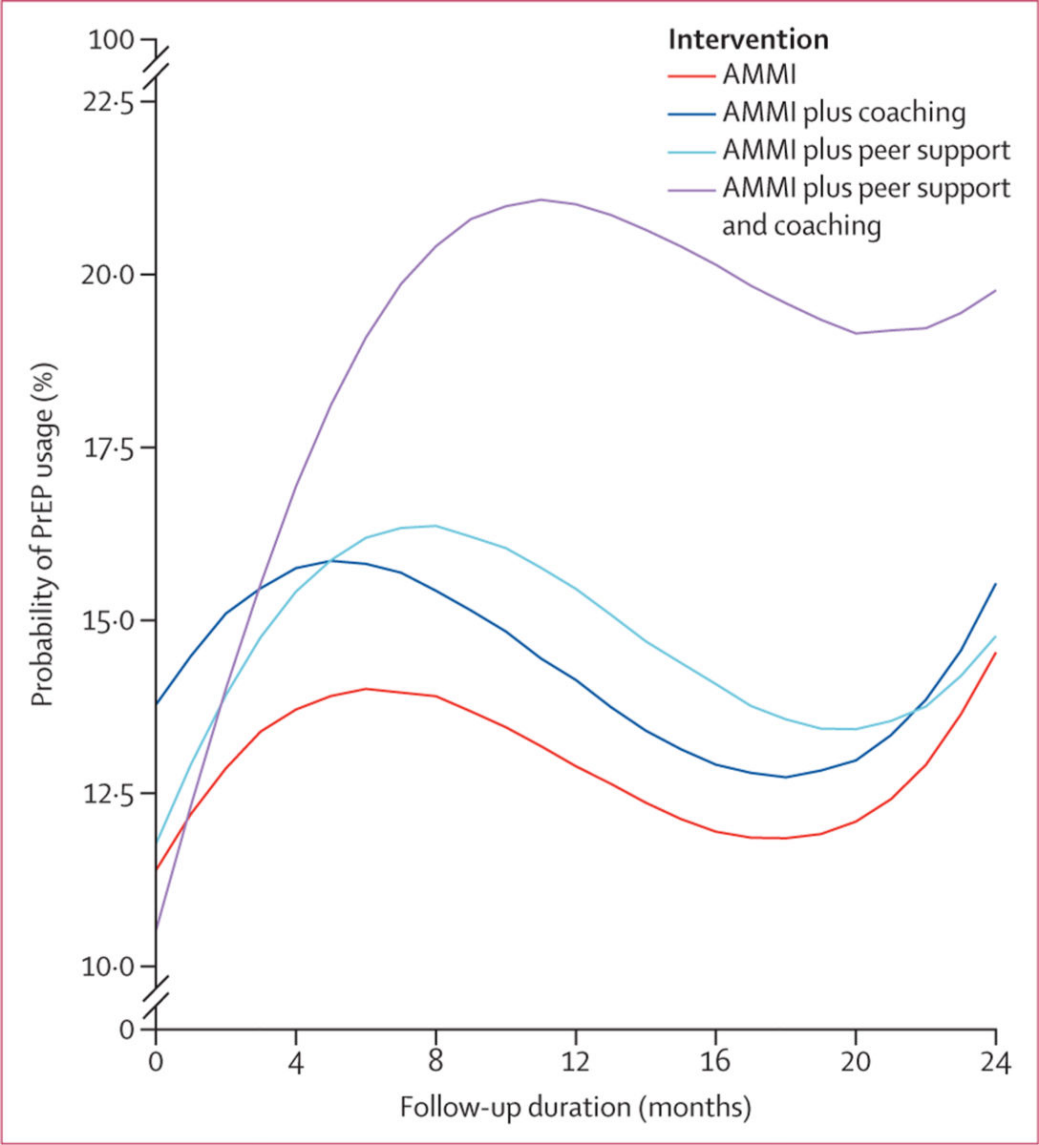
Predicted probabilities of PrEP usage over time by intervention group AMMI=automated text messaging and monitoring. PrEP=pre-exposure prophylaxis.

No significant changes were identified in consistent condom use with all partners between the AMMI only reference group and the AMMI plus coaching group (OR 0·95, 95% PI 0·86–1·04) and AMMI plus coaching and peer support group (0·95, 0·86–1·05), and although changes in the AMMI plus peer support group were significant at the 95% PI level (0·90, 0·82–0·99), they were not significant after adjusting for multiple comparisons (98·75% PI 0·81–1·03; appendix p 8). No significant changes were identified for PrEP adherence (ie, taking prescribed doses “all of the time” *vs* “most of the time” or less often) between the AMMI only reference group and the AMMI plus peer support group (OR 1·02, 95% PI 0·92–1·96), the AMMI plus coaching group (0·93, 0·71–1·20), and the AMMI plus coaching and peer support group (1·02, 0·78–1·32; appendix p 9). No significant changes were identified for number of recent sexual partners between the AMMI only reference group and the AMMI plus peer support group (0·99, 0·96–1·03), AMMI plus coaching group (1·03, 0·99–1·07), and the AMMI plus coaching and peer support group (1·01, 0·97–1·05; appendix p 8). No significant changes were identified for PEP prescription between the AMMI only reference group and the AMMI plus peer support group (1·01, 0·89–1·15), the AMMI plus coaching group (0·98, 0·86–1·11), and the AMMI plus coaching and peer support group (1·03, 0·89–1·15; appendix p 10). No significant changes were identified for PEP adherence (ie, “took all doses”) between the AMMI only reference group and the AMMI plus peer support group (1·25, 0·98–1·60), the AMMI plus coaching group (0·94, 0·74–1·17), and the AMMI plus coaching and peer support group (1·12, 0·89–1·40; appendix p 10). Table 2 shows regression coefficients for current PrEP use on the odds ratio (OR) scale with 95% and 98·75% posterior intervals (PIs) to account for multiple comparisons. We selected the GLMM that assumes cubic shapes across groups on the basis of widely applicable information criterion values and fitted to observed outcome trajectories shown in figure 2 (percentages are shown in the appendix [p 11]). Baseline current PrEP use was balanced across groups on the basis of OR PIs that include 1 for the three estimated coefficients for AMMI plus peer support, AMMI plus coaching, and AMMI plus peer support and coaching groups compared with the AMMI only group intercept (reference; table 2). Figure 3 shows predicted probabilities of PrEP use estimated from the regression model in table 2 that adjust for missing observations, time, enrolment dates, and the COVID-19 stay-at-home order beginning on March 17, 2020 (percentages are shown in the appendix [p 11]). Visually, we observed increased PrEP use for all groups from baseline to month 4, then decreasing for all groups except for the combined intervention group until around month 20, and increasing from months 20 to 24 (figure 3). There were higher rates in the combined intervention group until 12 months and some decrease from months 12 to 24, but PrEP use remained higher in this group than in the other intervention groups. The results in table 2 indicate that the increase between baseline and 4 months with visit (AMMI only reference) was significant (OR 2·15, 95% PI 1·26–3·72, 98·75% PI 1·08–4·30; table 2) and no differences for AMMI plus peer support and AMMI plus coaching. By contrast, AMMI plus peer support and coaching showed significantly higher PrEP use over time compared with AMMI only (and other groups), with the linear (visit) peer support plus coaching × time interaction coefficients representing trajectory differences relative to AMMI and PIs excluding 1 (OR 2·31, 95% PI 1·28–4·14, 98·75% PI 12–4·86, table 2). The quadratic (visit^2^) results statistically support the decrease in PrEP use shown in figures 2 and 3 after the initial increases (OR 0·72, 95% PI 0·59–0·88, 98·75% PI 0·56–0·92). AMMI plus peer support and coaching did not have a steeper slope of decline compared with AMMI only when adjusting for multiple comparisons (OR 0·89, 95% PI 0·82–0·98, 98·75% PI 0·79–1·01).

Table 3 presents regression coefficient estimates for the model adjusting for covariates. We reached the same conclusion that PrEP use increased and remained higher over time with AMMI plus peer support and coaching compared with AMMI alone, AMMI plus peer support, and AMMI plus coaching on the basis of the regression coefficients for the intervention conditions, time trajectories, and condition × time interactions. The overall trend in increased PrEP use for the AMMI reference group and coaching or peer support groups was not statistically significant for the linear (visit) result when adjusting for covariates and multiple comparisons (OR 1·89, 95% PI 1·01–3·56, 98·75% PI 0·86–4·30) but was higher for the peer support plus coaching group (2·75, 1·42–5·34, 1·22–6·45) compared with the model in table 2. Quadratic (visit^2^) estimates were similar in results and interpretation to the model without covariates (table 2). We also note that participants who were older, had insurance at baseline, had condomless sex with partners with HIV, and reported higher numbers of recent sex partners had increased odds of PrEP use at baseline. Participants identifying as bisexual (*vs* gay) or who had ever been incarcerated had decreased odds of using PrEP at baseline.

Results were similar for GLMMs and GEE models and analytical and baselined samples (data not shown).

No unanticipated study-related adverse events occurred.

## Discussion

In this study, the combined intervention condition of AMMI, online peer support, and telehealth coaching led to a significantly larger increase in PrEP use that was sustained over time than did AMMI only control and other conditions. Results are consistent with the study hypotheses that the combined intervention would have synergistic rather than additive effects^20^ due to complementary intervention functions. AMMI functioned to provide informational, motivational, resource, and reminder prompts for HIV prevention and related factors. Peer support provided opportunities for young people to share their experiences and query peers (eg, to demystify and normalise PrEP use). Coaching provided navigation with goal setting, problem solving, skills building, and follow-up accountability for young people to follow through on linkages for PrEP and other goals and services.

Results on significant increases in current PrEP use from baseline to 4 months for the AMMI only reference, peer support, and coaching groups mirror national trends for PrEP coverage (the ratio of users to those with indication for PrEP) of about 15% among young people aged 16–24 years in 2019–20.^1^ Observed and estimated PrEP use in the combined intervention reached greater than 20% of participants by 8 months (figures 2, 3; appendix p 11), approaching 2019 PrEP coverage rates that include older adults reported by the CDC for the nation (24·5%), California state (25·3%), and Louisiana state (24·6%).^1^ According to modelling studies by LeVasseur and colleagues,^29^ 25% PrEP coverage might be sufficient to bend the curve on new HIV infections in the absence of other prevention strategies among GBMSM. Furthermore, this coverage ratio exceeds the national average for Hispanic or Latinx people (14·5%) and Black or African American people (8·0%),^1^ groups that each make up 29% and 40% of our study sample, respectively. This difference suggests that the combined intervention might mitigate age and racial or ethnic disparities in PrEP use. Post-hoc analyses are in progress to assess subgroup intervention effects, including based on condom use and partner numbers given that there were no intervention effects on condom use, sexual partner numbers, PEP prescription, or adherence to PrEP or PEP.

There are several potential study limitations. First, the COVID-19 pandemic affected study procedures and possibly retention, numbers of sexual partners, and PrEP need and access.^1^ However, we controlled for COVID-19 onset in analyses and its effects would probably have affected all study groups, including the observed and predicted decreases and increases in PrEP use over the last three study assessments (figures 2, 3; appendix p 11). We also controlled for factors associated with loss to follow-up, given attrition over 2 years of follow-up. Second, the sample might not be representative of or generalisable to young people in other locations and settings. However, the sample is diverse and might be representative of young people reachable by prevention programmes as the recruitment methods parallel real-world practices. Third, the PrEP outcome is based on self-report of current use at each assessment and includes new, continued, and discontinued use over time. Because we were not embedded in PrEP clinics or pharmacies, we did not have access to electronic health record data for appointments or prescriptions. Furthermore, assessment of PrEP persistence within individuals is complicated by variability in PrEP indication over time, particularly among young people and during COVID-19 disruptions.^30^ Finally, the enhanced standard of care that all participants received of automated texting, repeat HIV and STI testing, same day bacterial STI treatment, follow-up and referrals by interviewers, and cash incentives for study visits are active interventions that do not reflect real-world standard of care. Intervention effects might be higher or lower compared with real-world standards of care, depending on assumptions regarding impacts of assessment effects and study procedures. The increases in PrEP use across all groups from baseline to 4 months might reflect intervention effects, assessment effects, historical trends, or a combination of these factors.

There is also a lot to consider on intervention participation (ie, dose), cost and cost-effectiveness, and implementation, which are beyond the scope of this Article. Briefly, the interventions are designed to be flexible for the diverse needs and preferences of young people, including intervention dose and methods (ie, texting, peer support, and coaching). About three-quarters of participants reported receiving text messages consistently, and more than half participated in coaching. Although only a quarter of young people participated in peer support, results suggest value for some young people in conjunction with other interventions. Future per-protocol or as-treated analyses of this trial’s data should account for anticipated heterogeneous needs, participation, and treatment effects given the broad inclusion criteria and diverse sample. Additionally, the interventions were designed to be easily adopted and adapted to diverse structural, community, and individual-level factors to support implementation, but varying staff efforts and costs are involved. We posited that coaching is the most costly and difficult intervention to implement but might still be cost-effective, depending on assumptions about implementation costs and costs saved. Online peer support required modest coach staff time and fees to sustain, but efforts were also needed to facilitate participation. In real-world practice, Discord would most likely be the preferred platform, which is free or $99 per year for premium hosting subscriptions. Text messaging is easily implementable but requires staff effort for follow-up on weekly monitoring survey responses. Many of the intervention functions could be automated with interactive tools, such as chatbots, which we explored with a small group of staff and participants from this study, suggesting that coach scheduling, referrals, round-the-clock availability, and tailored text messaging could be useful.^31^ Future analyses will examine implementation costs for all interventions in detail in conjunction with cost-effectiveness analyses and modelling medical and societal costs. Further research will examine adaptation and implementation barriers, facilitators, and opportunities for scale-up and sustainability for the interventions with community-based organisations, clinics, and public health stakeholders.^32^

This study shows that a combination of evidence-based strategies designed to be delivered via easily adoptable and adaptable technology platforms (ie, texting, online peer support, and telehealth) and paraprofessional front-line HIV prevention near-peers is efficacious in increasing PrEP use and sustained use over time among diverse populations of young people. The findings have implications for development of future interventions for young people at risk of HIV acquisition, including null results for condom use, sexual partner numbers, and PEP use and adherence. More research is needed to understand the associations between prevention option choice among young people with inconsistencies in risk behaviours over time.^30^ Future research should examine the interventions used in this study in implementation-effectiveness studies with front-line HIV prevention organisations and compare it with real-world standards of care.

## Supplementary Material

Supplementary material

## Figures and Tables

**Table 1:** Baseline characteristics for young people at risk for HIV in Los Angeles and New Orleans by intervention group and total analytic sample

	AMMI only (n=313)	AMMI plus peer support (n=205)	AMMI plus coaching (n=196)	AMMI plus peer support and coaching (n=181)	Overall (n=895)
Mean age, years (SD)	21·05 (2·07)	21·00 (2·27)	20·88 (2·19)	21·19 (2·12)	21·03 (2·15)
Sex					
Male	286 (91%)	194 (95%)	180 (92%)	170 (94%)	830 (93%)
Female	27 (9%)	11 (5%)	16 (8%)	11 (6%)	65 (7%)
Gender*					
Cisgender	249 (80%)	173 (84%)	147 (75%)	155 (86%)	724 (81%)
Transgender man	20 (6%)	9 (4%)	13 (7%)	6 (3%)	48 (5%)
Transgender woman	19 (6%)	15 (7%)	17 (9%)	8 (4%)	59 (7%)
Gender-diverse man	18 (6%)	6 (3%)	16 (8%)	7 (4%)	47 (5%)
Gender-diverse woman	7 (2%)	2 (1%)	3 (2%)	5 (3%)	17 (2%)
Sexual orientation†					
Gay	171 (55%)	121 (59%)	102 (52%)	107 (59%)	501 (56%)
Bisexual	78 (25%)	51 (25%)	56 (29%)	49 (27%)	234 (26%)
Pansexual	28 (9%)	9 (4%)	18 (9%)	11 (6%)	66 (7%)
Heterosexual	17 (5%)	8 (4%)	11 (6%)	5 (3%)	41 (5%)
Queer	12 (4%)	13 (6%)	6 (3%)	6 (3%)	37 (4%)
Other non-heterosexual	7 (2%)	3 (1%)	3 (2%)	3 (2%)	16 (2%)
Race or ethnicity					
Black or African American‡	141 (45%)	79 (39%)	77 (39%)	65 (36%)	362 (40%)
Latinx or Hispanic	80 (26%)	58 (28%)	61 (31%)	58 (32%)	257 (29%)
White (non-Hispanic)	60 (19%)	47 (23%)	33 (17%)	44 (24%)	184 (21%)
Asian or Pacific Islander	20 (6%)	14 (7%)	10 (5%)	9 (5%)	53 (6%)
Other or mixed background	12 (4%)	7 (3%)	15 (8%)	5 (3%)	39 (4%)
City					
Los Angeles, CA, USA	185 (59%)	127 (62%)	120 (61%)	114 (63%)	546 (61%)
New Orleans, LA, USA	128 (41%)	78 (38%)	76 (39%)	67 (37%)	349 (39%)
Education					
Below high school	55 (18%)	30 (15%)	28 (14%)	24 (13%)	137 (15%)
High school or equivalent	77 (25%)	41 (20%)	47 (24%)	41 (23%)	206 (23%)
Some higher education	144 (46%)	94 (46%)	100 (51%)	81 (45%)	419 (47%)
Completed higher education	34 (11%)	35 (17%)	19 (10%)	31 (17%)	119 (13%)
Income above federal poverty level (2021)	101 (32%)	74 (36%)	79 (40%)	54 (30%)	308 (34%)
Insurance status					
Insured	235 (75%)	166 (81%)	147 (75%)	139 (77%)	687 (77%)
Uninsured or unsure	78 (25%)	39 (19%)	49 (25%)	42 (23%)	208 (23%)
Sexual risk and protective behaviours					
Sexually transmitted infection (in lifetime)	104 (33%)	71 (35%)	69 (35%)	64 (35%)	308 (34%)
Sexually transmitted infection (in past 4 months)	78 (25%)	46 (22%)	49 (25%)	48 (27%)	221 (25%)
Condomless sex with partner with HIV (in lifetime)§	27/305 (9%)	16/192 (8%)	14/184 (8%)	19/176 (11%)	76/857 (9%)
Condomless anal sex in past year	212 (68%)	120 (59%)	131 (67%)	128 (71%)	591 (66%)
100% condom use with all partners (in lifetime)§	67/305 (22%)	49/192 (26%)	30/184 (16%)	30/176 (17%)	176/857 (21%)
100% condom use with all partners (in past 4 months)¶	118/275 (43%)	72/173 (42%)	63/163 (39%)	58/157 (37%)	311/768 (40%)
No sexual activity (in past 4 months)	38 (12%)	32 (16%)	33 (17%)	24 (13%)	127 (14%)
Mean number of recent sexual partners (in past 4 months; SD)	3·70 (6·99)	5·46 (19·25)	3·09 (3·95)	5·06 (15·66)	4·25 (12·51)
PrEP use (in lifetime)	49 (16%)	38 (19%)	44 (22%)	36 (20%)	167 (19%)
PrEP use (current)	34 (11%)	23 (11%)	26 (13%)	18 (10%)	101 (11%)
PEP use (in lifetime)*	15 (5%)	6 (3%)	17 (9%)	15 (8%)	53 (6%)
PEP use (in past 4 months)*	4 (1%)	3 (1%)	8 (4%)	9 (5%)	24 (3%)
Sex exchange (in lifetime)*	93 (30%)	43 (21%)	40 (20%)	41 (23%)	217 (24%)
Substance use					
Cannabis use (in past 4 months)	231 (74%)	140 (68%)	142 (72%)	135 (75%)	648 (72%)
AUDIT-C hazardous drinking	126 (40%)	93 (45%)	78 (40%)	73 (40%)	370 (41%)
Opioid use (in past 4 months)	13 (4%)	13 (6%)	10 (5%)	14 (8%)	50 (6%)
Stimulant use (cocaine or methamphetamines)	69 (22%)	49 (24%)	47 (24%)	42 (23%)	207 (23%)
Poppers (nitrite inhalants) use (in lifetime)*	74 (24%)	68 (33%)	58 (30%)	60 (33%)	260 (29%)
Mental health					
Suicide attempt (in lifetime)	103 (33%)	50 (24%)	63 (32%)	51 (28%)	267 (30%)
PHQ-9 depression symptoms (clinical cutoff)	98 (31%)	62 (30%)	57 (29%)	55 (30%)	272 (30%)
GAD-7 anxiety symptoms (clinical cutoff)	124 (40%)	70 (34%)	63 (32%)	65 (36%)	322 (36%)
Mental health hospitalisation (in lifetime)*	94 (30%)	36 (18%)	45 (23%)	31 (17%)	206 (23%)
Homelessness (in lifetime)*	140 (45%)	58 (28%)	57 (29%)	62 (34%)	317 (35%)
Incarceration (in lifetime)*	62 (20%)	26 (13%)	23 (12%)	30 (17%)	141 (16%)
Intimate partner violence (in lifetime)*	108 (35%)	57 (28%)	59 (30%)	49 (27%)	273 (31%)
Support services use (in past 4 months)*	158 (50%)	83 (40%)	60 (31%)	57 (31%)	358 (40%)

Data are n (%) or n/N (%) unless otherwise stated. Some percentages do not total 100% due to missing values. AMMI=automated text messaging and monitoring. AUDIT-C=alcohol use disorders identification test consumption. PrEP=pre-exposure prophylaxis. PEP=post-exposure prophylaxis. PHQ-9=Patient Heath Questionnaire-9. GAD-7=Generalized Anxiety Disorder-7.

* χ^2^ tests of independence for imbalances at baseline between groups p<0·05.

† Sexual orientation is given as was reported at baseline. Men who have sex with men identifying as heterosexual were classified as gay or bisexual on the basis of behavioural reports for the longitudinal analysis.

‡ Includes young people specifying Black non-Hispanic and 64 people reporting Black Hispanic or Black and other or mixed background, where other or mixed background excludes Black or African American.

§ Denominators exclude missing data, refusals, and participants not reporting vaginal or anal intercourse in their lifetime.

¶ Denominators exclude participants reporting no sexual activity in past 4 months at baseline.

**Table 2:** Regression results in the odds scale for current PrEP use over time by variable

	OR estimate	95% PI	98·75% PI
Intercept	0·01	0·00–0·01	0·00–0·01
AMMI plus coaching group	1·73	0·57–5·57	0·42–7·75
AMMI plus peer support group	1·14	0·38–3·44	0·29–4·63
AMMI plus peer support and coaching group	0·88	0·27–2·92	0·20–4·08
Visit	2·15	1·26–3·72	1·08–4·30
Visit × AMMI plus coaching group	0·89	0·50–1·58	0·43–1·90
Visit × AMMI plus peer support group	1·30	0·75–2·30	0·64–2·69
Visit × AMMI plus peer support and coaching group	2·31	1·28–4·14	1·12–4·86
Visit^2^	0·72	0·59–0·88	0·56–0·92
Visit^2^ × AMMI plus coaching group	1·01	0·92–1·11	0·90–1·14
Visit^2^ × AMMI plus peer support group	0·96	0·87–1·04	0·85–1·07
Visit^2^ × AMMI plus peer support and coaching group	0·89	0·82–0·98	0·79–1·01
Visit^3^	1·04	1·02–1·06	1·01–1·07

The reference group is AMMI only. Visit is the time variable of these data where time between each visit is about 4 months. Visit is linear, Visit^2^ is quadratic, and Visit^3^ is cubic. The 98·75% PI is based on conservative Bonferroni correction across the four primary outcomes. AMMI=automated text messaging and monitoring. OR=odds ratio. PI=posterior interval. PrEP=pre-exposure prophylaxis.

**Table 3:** Regression results in odds scale for current PrEP use over time controlling for covariates by variable

	OR estimate	95% PI	98·75% PI
Intercept	0·00	0·00–0·00	0·00–0·00
AMMI plus coaching group	1·73	0·49–6·31	0·33–9·15
AMMI plus peer support group	0·90	0·26–3·00	0·19–4·25
AMMI plus peer support and coaching group	0·69	0·20–2·39	0·14–3·45
Visit	1·89	1·01–3·56	0·86–4·30
Visit × AMMI plus coaching group	0·99	0·51–1·91	0·42–2·31
Visit × AMMI plus peer support group	1·59	0·84–2·99	0·71–3·50
Visit × AMMI plus peer support and coaching group	2·75	1·42–5·34	1·22–6·45
Visit^2^	0·76	0·60–0·95	0·56–1·01
Visit^2^ × AMMI plus coaching group	0·99	0·89–1·09	0·86–1·13
Visit^2^ × AMMI plus peer support group	0·93	0·84–1·03	0·82–1·05
Visit^2^ × AMMI plus peer support and coaching group	0·87	0·79–0·97	0·76–0·99
Visit^3^	1·03	1·01–1·06	1·00–1·07
Age	1·26	1·06–1·51	1·01–1·58
Gender*			
Cisgender	1 (ref)	··	··
Transgender or gender diverse	2·50	0·83–7·67	0·61–10·28
Race or ethnicity			
White (non-Hispanic)	1 (ref)	··	··
Black or African American	1·37	0·51–4·08	0·39–5·73
Latinx or Hispanic	1·27	0·47–3·60	0·35–4·98
Other†	1·36	0·36–5·27	0·23–7·82
Sexual orientation*			
Gay	1 (ref)	··	··
Bisexual	0·22	0·09–0·56	0·07–0·72
Other	0·53	0·16–1·72	0·11–2·35
City			
Los Angeles, CA, USA	1 (ref)	··	··
New Orleans, LA, USA	1·81	0·75–4·23	0·59–5·33
Insured	4·06	2·47–6·79	2·19–7·90
Income above federal poverty level (2021)	1·90	0·89–3·92	0·72–4·94
Condomless anal sex in the past year	1·20	0·55–2·63	0·44–3·30
Homelessness (in lifetime)	0·37	0·14–0·93	0·11–1·18
Incarceration (in lifetime)	0·20	0·06–0·64	0·04–0·89
Popper use (in lifetime)	2·50	1·10–5·78	0·82–7·42
Sex exchange (in lifetime)	0·91	0·35–2·27	0·28–2·84
Condomless sex with partner with HIV (in lifetime)	17·43	5·74–55·24	4·23–75·44
Number of recent sexual partners (in past 4 months)‡	3·03	2·32–3·98	2·16–4·30
Intimate partner violence (in lifetime)	0·87	0·39–1·90	0·31–2·48
Used support services in past 4 months	0·94	0·62–1·44	0·56–1·60
After COVID-19§	0·90	0·54–1·48	0·47–1·71
Enrolment date¶	0·93	0·75–1·15	0·71–1·22

The 98·75% PI is based on conservative Bonferroni correction across the four primary outcomes. Visit is linear, Visit^2^ is quadratic, and Visit^3^ is cubic. AMMI=automated text messaging and monitoring. OR=odds ratio. PI=posterior interval. PrEP=pre-exposure prophylaxis.

* Some gender and sexual orientation response categories shown in table 1 were combined for longitudinal analyses due the small proportion of people in some categories.

† Asian, Hawaiian or Pacific Islander, native American, Alaskan native, and other are combined due to the small proportion of people in some categories.

‡ The cubed root of this variable was taken to ameliorate positive skew.

§ Follow-up visit occurred after March 16, 2020.

¶ Centred at midpoint enrolment time and scaled by 100 days.

## Research in context

### Evidence before this study

We conducted literature searches from Dec 2, 2015, to Jan 18, 2016, and an updated search before the protocol change from July 20 to Aug 10, 2018. We searched for systematic literature reviews, meta-analyses, and individual studies on Web of Science, PubMed, and PsychINFO on coaching or counselling, peer support, social media, mobile health, and texting interventions. We included search terms “HIV”, “PrEP”, “pre-exposure prophylaxis”, “PEP”, “postexposure prophylaxis”, “sexual health”, “condom”, “HIV prevention”; “coaching”, “coach”, “peer support”, “peer”, “group”, “peer navigation”, “text”, “SMS”, “mobile phone”, “cell phone”, “technology”, “text messaging”, “text message”, “youth”, “adolescent”, “young adult”, “GBMSM”, “gay”, “LGBT”, “LGBTQ”, “MSM”, “men who have sex with men”, “intervention”, “program”, “programme”, “project”, and “trial”. We also reviewed articles from the US Centers for Disease Control and Prevention (CDC) PrEP Best Practices Intervention List and Risk Reduction Evidence Based Interventions from the Prevention Research Synthesis Project. Only English articles were included. Articles were excluded if they were published before 2000, did not report on relevant outcomes, did not report odds ratios or effect size data, or did not use a relevant intervention method. To date, the CDC has identified two evidence-based and two evidence-informed (ie, promising evidence) interventions improving pre-exposure prophylaxis (PrEP) uptake for sexual and gender minority populations in the USA, but the interventions were brief with duration of effects or follow-up of 3 months or less. Despite disproportionately high HIV incidence and prevalence among young people of sexual and gender minorities, PrEP uptake is low. At study conception in 2016, there were no evidence-based interventions showing efficacy in PrEP uptake. There was some evidence for effects on other HIV prevention outcomes (ie, HIV testing, condom use, and sexual partner strategies) for interventions using automated text messaging informational and motivational prompts, online peer forums, and counselling or coaching methods. However, most evidence-based interventions are not scaled up, not delivered with fidelity to manuals, or not sustained in real-world practice.

### Added value of this study

This study provides evidence that disruptive innovation approaches of simpler, flexible, and adaptable interventions designed for diffusion (ie, easily adoptable and adaptable) that account for diverse and competing needs and syndemic factors can improve PrEP uptake. This is in contrast to current norms for highly scripted and brief interventions narrowly targeting HIV prevention or PrEP uptake that do not provide ongoing support during varying periods of risk and developmental transitions.

### Implications of all the available evidence

This study provides evidence on the impact of an intervention package that is designed for adoption, adaptation, and implementation by front-line HIV prevention workers to improve and sustain PrEP use among diverse youth.
