# Supplementary material for: Optimal strategies to improve uptake of and adherence to HIV prevention among young people at risk for HIV acquisition in the USA (ATN 149): a randomised, controlled, factorial trial
Source: Lancet Digit Health. Author manuscript; Available in PMC 2024 Sep 3. (PMC11371121; doi:10.1016/S2589-7500(23)00252-2)
Supplement: Supplementary material [file NIHMS2002000-supplement-Supplementary_material.pdf]

THE LANCET
Digital Health

Supplementary appendix

This appendix formed part of the original submission and has been peer reviewed. We post it as supplied by the authors.

Supplement to: Swendeman D, Rotheram-Borus MJ, E Mayfield Arnold E, et al. Optimal strategies to improve uptake of and adherence to HIV prevention among young people at risk for HIV acquisition in the USA (ATN 149): a randomised, controlled, factorial trial. *Lancet Digit Health* 2024; **6:** e187–200.

Supplemental Materials Appendices – Swendeman et al., ATN 149

ATN CARES and ATN 149 study group authors

USA S E Abdalian (Tulane University School of Medicine, New Orleans, LA); R Bolan, R Flynn (Los Angeles LGBT Center, Los Angeles, CA); Y Bryson, R Cortado, T Kerin, K Nielsen, N Milburn, W Ramos, R E Weiss (University of California, Los Angeles, CA); J Klausner, P H Rezvan (University of Southern California, Los Angeles, CA); M Lightfoot (Oregon Health and Science University-Portland State University School of Public Health, Portland, OR); W Tang (Harvard University, Cambridge, MA)

Supplemental Figure 1: Proportions of Coaching Sessions Using Evidence-Based Practice Elements Delivered by Coaches as Documented in Study Case Management System

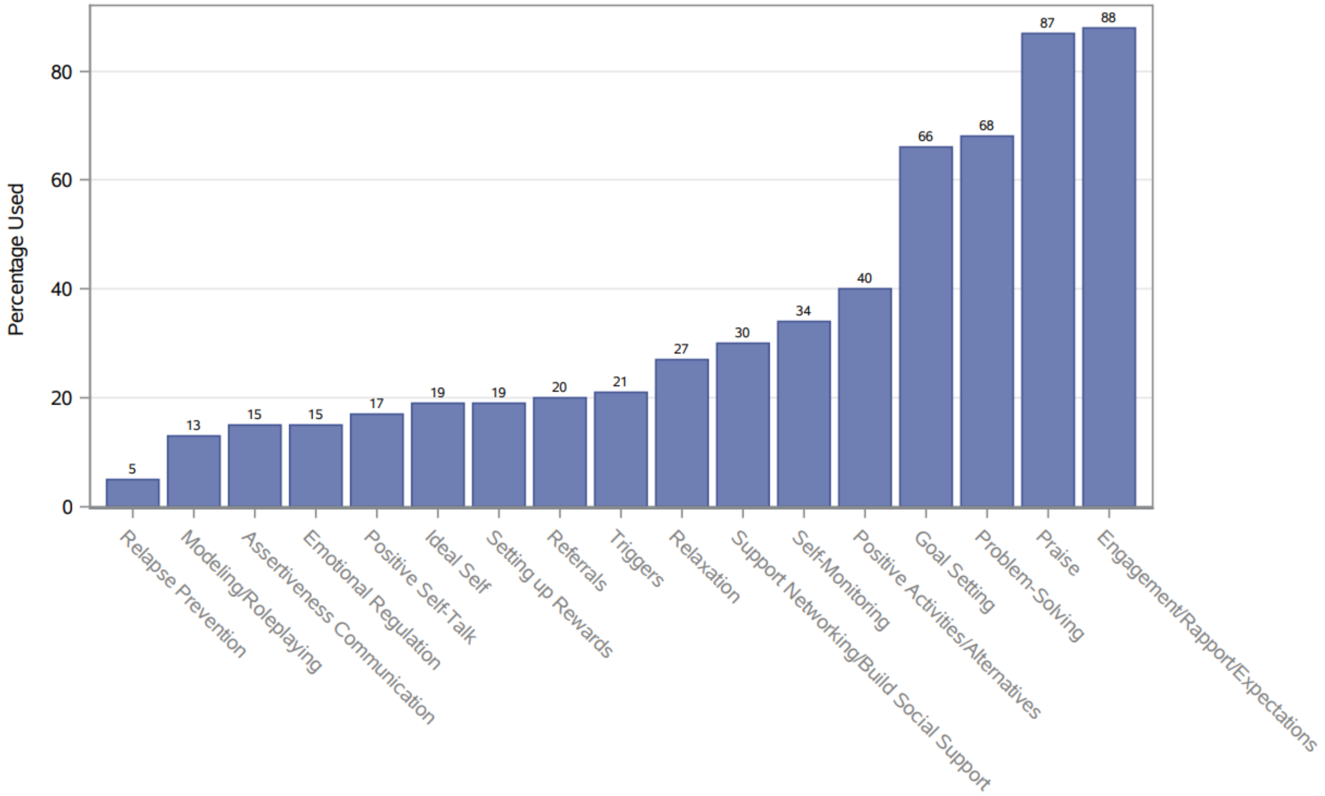

Supplemental Figure 2: Proportions of Coaching Sessions Covering Contents Areas Discussed by Coaches as Documented in Study Case Management System

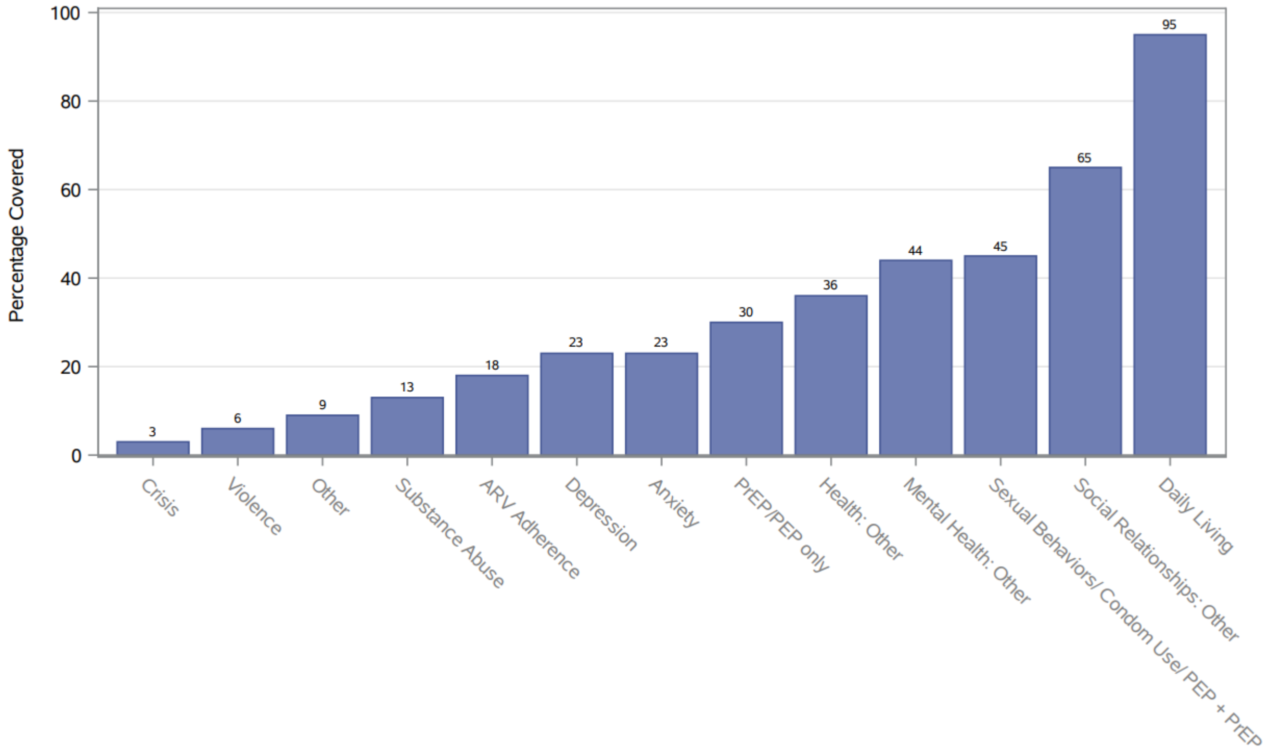

Supplemental Table 1: Numbers and Percentages of Study Visits Completed by Arm (N = 895)

# of Visits	AMMI	AMMI + Coach	AMMI + PS	AMMI + PS + Coach
1	24 (7.67%)	12 (6.12%)	10 (4.88%)	11 (6.08%)
2	27 (8.63%)	11 (5.61%)	13 (6.34%)	19 (10.5%)
3	19 (6.07%)	17 (8.67%)	27 (13.17%)	16 (8.84%)
4	32 (10.22%)	30 (15.31%)	13 (6.34%)	20 (11.05%)
5	58 (18.53%)	39 (19.9%)	47 (22.93%)	33 (18.23%)
6	153 (48.88%)	87 (44.39%)	95 (46.34%)	82 (45.3%)

Notes: PS = Peer Support. Chi-squared test for independence indicate no significant association between number of follow-up visits and intervention arm (χ^2=22.169, df=15, p=0.1034). Percentages are based on number of participants in each arm (column percentages), for example, the AMMI+Coaching arm had 12 participants with only one follow-up visit; these 12 participants make up 6.12% of the total number of participants in the AMMI+Coaching group. Supplemental Figure 3 below shows number of study visits across all study arms.

Supplemental Figure 3. Number of Follow-Up Study Visits Completed (n=895).

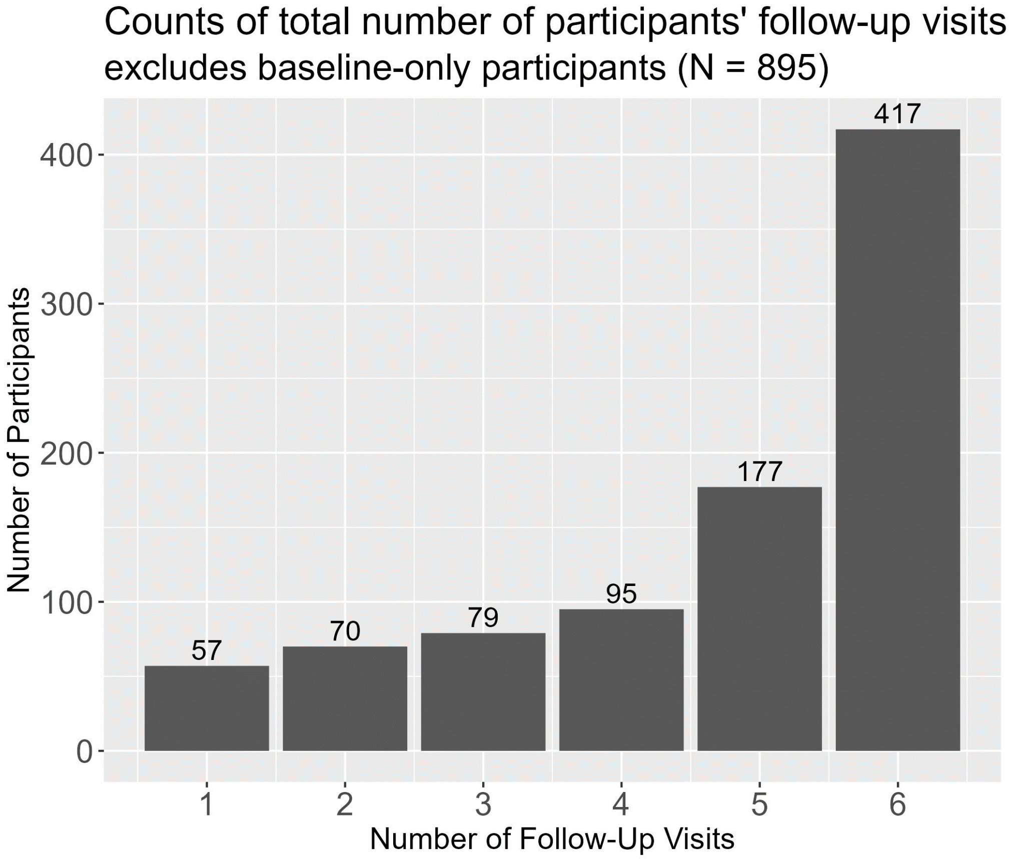

Supplemental Table 2 and Supplemental Figure 4 show further elaborates that there is little difference in retention across arms.

Supplemental Table 2. Retention of Participants in Each Study Intervention Arm Over Time

	**Months Since Baseline**						
**Arm**	**0**	**4**	**8**	**12**	**16**	**20**	**24**
AMMI	313 (100%)	288 (92%)	269 (85.9%)	246 (78.6%)	232 (74.1%)	213 (68.1%)	223 (71.2%)
AMMI + Coach	196 (100%)	165 (84.2%)	164 (83.7%)	163 (83.2%)	146 (74.5%)	149 (76%)	135 (68.9%)
AMMI + Peer Support	205 (100%)	187 (91.2%)	174 (84.9%)	162 (79%)	156 (76.1%)	145 (70.7%)	150 (73.2%)
AMMI + Peer Support + Coach	181 (100%)	165 (91.2%)	157 (86.7%)	138 (76.2%)	132 (72.9%)	125 (69.1%)	117 (64.6%)
Total	895 (100%)	805 (89.9%)	764 (85.4%)	709 (79.2%)	666 (74.4%)	632 (70.6%)	625 (69.8%)

Percents are based on total number of participants in each arm at baseline.

Supplemental Figure 4. Retention of Participants in Each Intervention Arm Over Time

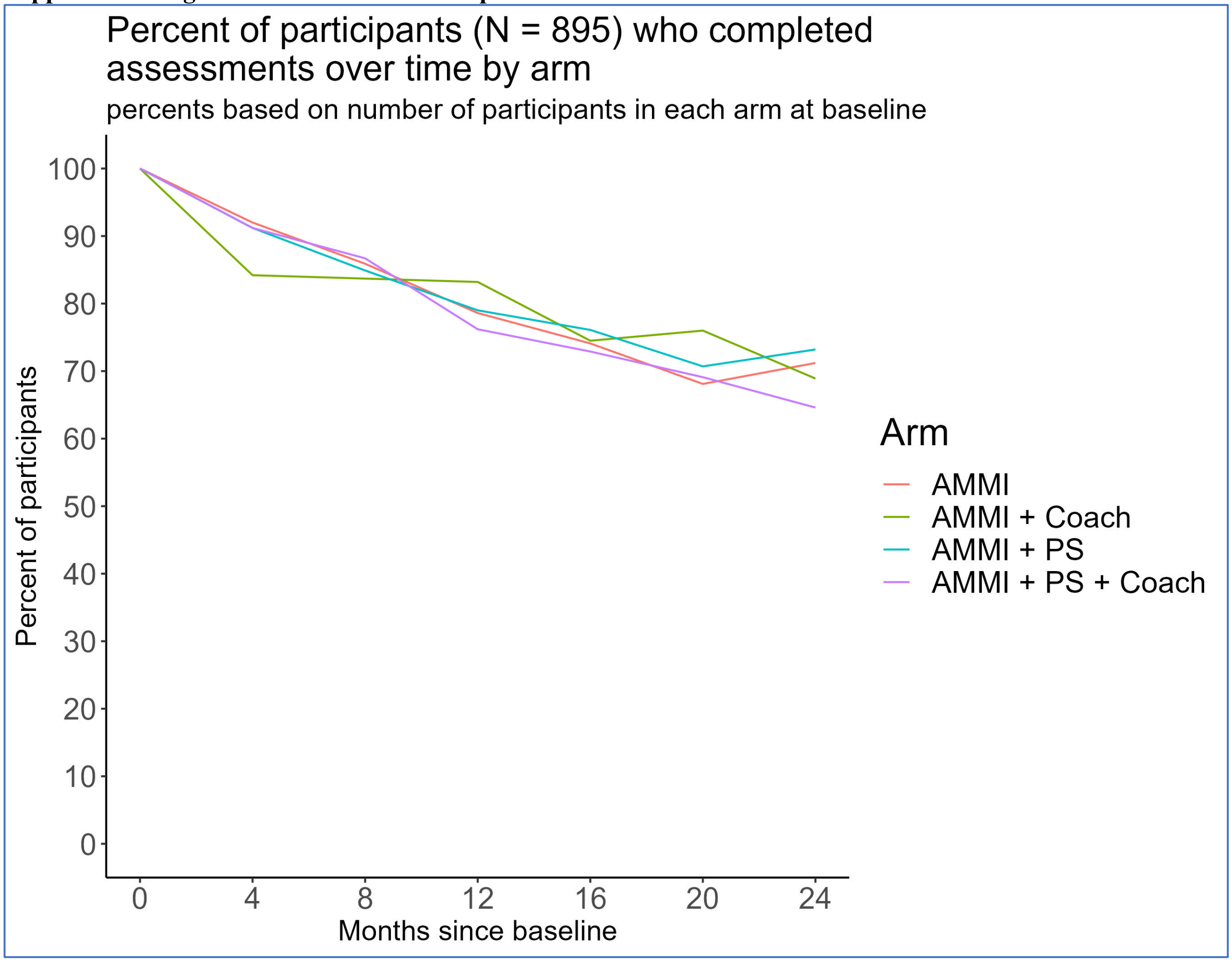

Supplemental Table 7 compares baseline characteristics of participants with follow-up in their second study year versus those who only had follow-up visit in year 1 (i.e. follow-up before their second study year). Using this dichotomized metric for loss-to-follow-up, we again find no statistically significant difference in retention across arms. There are differences comparing year 1 and 2 follow-up visit groups; participants with no year 2 follow-up tended to be less educated, have used cannabis recently, have not used PrEP recently or in their lifetime, been hospitalized for mental health in their lifetime, or been homeless or incarcerated before.

Supplemental Table 3: Baseline Characteristics of Participants with and without Follow-up Visits in Their Second Year of the Study

	Has 2nd year follow-up (N=752)	No 2nd year follow-up (N=143)	Overall (N=895)	P-value
**Arm**				
AMMI	260 (34.6%)	53 (37.1%)	313 (35.0%)	0.317
AMMI + Coach	171 (22.7%)	25 (17.5%)	196 (21.9%)	
AMMI + Peer Support	175 (23.3%)	30 (21.0%)	205 (22.9%)	
AMMI + Peer Support + Coach	146 (19.4%)	35 (24.5%)	181 (20.2%)	
**Age (years)**				
Mean (SD)	21.0 (2.16)	20.9 (2.14)	21.0 (2.15)	0.578
**Sex**				
Female	56 (7.4%)	9 (6.3%)	65 (7.3%)	0.756
Male	696 (92.6%)	134 (93.7%)	830 (92.7%)	
**Gender**				
Cisgender	609 (81.0%)	115 (80.4%)	724 (80.9%)	0.83
Gender diverse Female	14 (1.9%)	3 (2.1%)	17 (1.9%)	
Gender diverse Male	37 (4.9%)	10 (7.0%)	47 (5.3%)	
Transgender Female	50 (6.6%)	9 (6.3%)	59 (6.6%)	
Transgender Male	42 (5.6%)	6 (4.2%)	48 (5.4%)	
**Sexual orientation**				
Bisexual	192 (25.5%)	42 (29.4%)	234 (26.1%)	0.173
Gay/Same Gender Loving/Downe	435 (57.8%)	71 (49.7%)	506 (56.5%)	
Other	124 (16.5%)	30 (21.0%)	154 (17.2%)	
**Race**				
Asian / Pacific Islander	47 (6.3%)	6 (4.2%)	53 (5.9%)	0.0756
Black / African American[++]	297 (39.5%)	65 (45.5%)	362 (40.4%)	
Latino	228 (30.3%)	29 (20.3%)	257 (28.7%)	
Other	33 (4.4%)	6 (4.2%)	39 (4.4%)	
White	147 (19.5%)	37 (25.9%)	184 (20.6%)	
**City**				
Los Angeles	464 (61.7%)	82 (57.3%)	546 (61.0%)	0.375
New Orleans	288 (38.3%)	61 (42.7%)	349 (39.0%)	
**Education***				
Below high school	111 (14.8%)	26 (18.2%)	137 (15.3%)	< .001

	Has 2nd year follow-up (N=752)	No 2nd year follow-up (N=143)	Overall (N=895)	P-value
High school/equivalent	153 (20.3%)	53 (37.1%)	206 (23.0%)	
Some higher education	369 (49.1%)	50 (35.0%)	419 (46.8%)	
Completed higher education	108 (14.4%)	11 (7.7%)	119 (13.3%)	
**Income**				
Above 2021 Federal poverty level	267 (35.5%)	41 (28.7%)	308 (34.4%)	0.139
**Insurance status**				
Insured	582 (77.4%)	105 (73.4%)	687 (76.8%)	0.357
Uninsured/Unsure	170 (22.6%)	38 (26.6%)	208 (23.2%)	
**Sexual Risk and Protective Behaviors**				
Sexually transmitted infection (Lifetime)	264 (35.1%)	44 (30.8%)	308 (34.4%)	0.372
Sexually transmitted infection (Recent)	190 (25.3%)	31 (21.7%)	221 (24.7%)	0.435
Condomless Sex with HIV+ partner (LF)	64 (8.5%)	12 (8.4%)	76 (8.5%)	1
Condomless anal sex in the past year	494 (65.7%)	97 (67.8%)	591 (66.0%)	0.714
100% Condom Use All Partners (Lifetime)	171 (22.7%)	41 (28.7%)	212 (23.7%)	0.174
100% Condom Use All Partners (past 4-months)	360 (47.9%)	78 (54.5%)	438 (48.9%)	0.188
No Sexual Activity (past 4-months)	107 (14.2%)	20 (14.0%)	127 (14.2%)	1
Number of recent sex parters				
Mean (SD)	4.21 (13.0)	4.49 (9.43)	4.25 (12.5)	0.806
PrEP Use (Lifetime)*	153 (20.3%)	14 (9.8%)	167 (18.7%)	0.0044
PrEP Use (past 4-months)*	97 (12.9%)	4 (2.8%)	101 (11.3%)	<.001
PEP Use (Lifetime)	48 (6.4%)	5 (3.5%)	53 (5.9%)	0.258
Completed all doses of PEP Last Time	36 (4.8%)	4 (2.8%)	40 (4.5%)	1
Sex exchange (Lifetime)	180 (23.9%)	37 (25.9%)	217 (24.2%)	0.689
**Substance Use**				
Cannabis use (past 4 months)*	530 (70.5%)	118 (82.5%)	648 (72.4%)	0.0053

	Has 2nd year follow-up (N=752)	No 2nd year follow-up (N=143)	Overall (N=895)	P-value
Audit-C Hazardous Drinking	318 (42.3%)	52 (36.4%)	370 (41.3%)	0.249
Opioid Use (past 4-months)	41 (5.5%)	9 (6.3%)	50 (5.6%)	0.835
Stimulants Use (past 4-months)	174 (23.1%)	33 (23.1%)	207 (23.1%)	1
Poppers use (Lifetime)	222 (29.5%)	38 (26.6%)	260 (29.1%)	0.562
**Mental Health**				
Suicide Attempt (Lifetime)	224 (29.8%)	43 (30.1%)	267 (29.8%)	0.994
PHQ-9 Depression Symptoms (clinical cutoff)	219 (29.1%)	53 (37.1%)	272 (30.4%)	0.072
GAD-7 Anxiety Symptoms (clinical cutoff)	263 (35.0%)	59 (41.3%)	322 (36.0%)	0.151
Mental Health Hosptialization (Lifetime)*	163 (21.7%)	43 (30.1%)	206 (23.0%)	0.0378
**Homelessness (Lifetime)***	241 (32.0%)	76 (53.1%)	317 (35.4%)	<.001
**Incarceration (Lifetime)***	105 (14.0%)	36 (25.2%)	141 (15.8%)	0.0011
**Interpersonal violence (Lifetime)**	220 (29.3%)	53 (37.1%)	273 (30.5%)	0.0659
**Service usage (past 4-months)***	284 (37.8%)	74 (51.7%)	358 (40.0%)	0.0027

Note: Some percentages do not total 100% due to missing values.
P-values are from the chi-squared test for independence for categorical variables, and from two sample t-test for continuous variables.
*Chi-squared tests of independence for imbalances at baseline between arms p<0.05
++Includes youth specifying both Black-non-Hispanic and Black-Hispanic

Supplemental Table 4: Regression Results for Condom Usage with All Partners in the Odds Scale by Intervention Arm and Time

Variable	Odds Ratio Estimate	95% Posterior Interval for OR
Intercept	0.34	0.25 – 0.45 *
Coaching Arm	1.36	0.85 – 2.17
Peer Support Arm	1.54	0.97 – 2.41
Peer Support + Coaching Arm	1.03	0.64 – 1.69
Visit	1.25	1.10 – 1.43
Visit × Coaching Arm	0.95	0.86 – 1.04
Visit × Peer Support Arm	0.90	0.82 – 0.99 *
Visit × Peer Support + Coaching Arm	0.95	0.86 – 1.05
$Visit^2$	0.98	0.96 – 1.00

Notes: Reference arm is the AMMI-only. Visit is the time variable where time between each visit is about 4 months. *Indicates that the odds ratio does not contain 1. Model selection was evaluated using WAIC (widely applicable information criterion), which indicated that a quadratic model without interactions between time and intervention arm had the best fit. The only intervention effect where the posterior interval for the odds ratio does not contain one is the peer support-visit interaction, which is very close to being insignificant since the upper limit of the 95% posterior interval is 0.99 and very close to 1, and is not statistically significant when using the multiple comparisons adjustment posterior interval (98.75% 0.81-1.03).

Variable	Estimate	95% Posterior Interval
Intercept	2.89	2.55 – 3.27 *
Coaching Arm	0.81	0.66 – 0.99 *
Peer Support Arm	0.99	0.81 – 1.21
Peer Support+Coaching Arm	1.04	0.84 – 1.27
Visit	0.90	0.88 – 0.92 *
Visit × Coaching Arm	1.03	0.99 – 1.07
Visit × Peer Support Arm	0.99	0.96 – 1.03
Visit × Peer Support+Coaching Arm	1.01	0.97 – 1.05

Supplemental Table 5: Regression Results for Number of Recent Sex Partners by Intervention Arm and Time

Notes: * Indicates that the posterior interval does not contain 1. Reference arm is the AMMI arm.

Notes: Estimates reported in table are exponentiated forms of the regression coefficients. The distribution of number of recent sex partners had a severe positive skew (median = 2, mean = 3.18, SD = 8.09, max = 300). Furthermore, about 20.6% of observations were zero. Therefore, we fit zero-inflated negative binomial and zero-inflated Poisson models. WAIC indicated that the zero-inflated negative binomial model with linear time provided the best fit to the data.

Tables 6a to 6c below show PrEP adherence regression results over time by study arm based on two questions with dichotomized responses and means for a pseudo-continuous 0 to 5 Likert response scale. Results demonstrate no statistically significant differences between study arms at baseline or over time. WAIC indicated models with linear time (visit) provided the best model fit.

Supplemental Table 6a: Regression Results for PrEP Adherence (Every Day vs. Almost Every Day or Less) by Intervention Arm and Time

Variable	Odds Ratio Estimate	95% Posterior Interval for OR
Intercept	3.53	1.72-7.60*
Coaching Arm	2.20	0.66-7.72
Peer Support Arm	0.90	0.28-2.80
Peer Support+Coaching Arm	1.39	0.40-4.83
Visit	0.98	0.81-1.18
Visit × Coaching Arm	0.92	0.68-1.24
Visit × Peer Support Arm	1.19	0.88-1.60
Visit × Peer Support+Coaching Arm	1.06	0.78-1.45

Supplemental Table 6b: Regression Results for PrEP Adherence (How much of the time taking past 30 days - All of the Time vs. Most of the Time or Less) by Intervention Arm and Time

Variable	Odds Ratio Estimate	95% Posterior Interval for OR
Intercept	0.91	0.46-1.80
Coaching Arm	2.08	0.73-6.11
Peer Support Arm	0.63	0.22-1.77
Peer Support+Coaching Arm	1.61	0.55-4.64
Visit	0.98	0.83-1.16
Visit × Coaching Arm	0.93	0.71-1.20
Visit × Peer Support Arm	1.20	0.92-1.56
Visit × Peer Support+Coaching Arm	1.02	0.78-1.32

Supplemental Table 6c: Regression Results for Mean PrEP Adherence (All of the Time=5 to None of the time=0) by Intervention Arm and Time

Variable	Estimate	95% Posterior Interval
Intercept	4.29	3.99-4.59*
Coaching Arm	-0.09	-0.54-0.35
Peer Support Arm	-0.11	-0.57-0.35
Peer Support+Coaching Arm	-0.04	-0.49-0.42
Visit	-0.05	-0.12-0.02
Visit × Coaching Arm	0.02	-0.09-0.13
Visit × Peer Support Arm	0.03	-0.08-0.14
Visit × Peer Support+Coaching Arm	0.04	-0.08-0.15

Tables 7a and 7b show PEP prescription and adherence (taking all doses) regression results over time and by study arms. Results demonstrate no statistically significant differences between study arms. WAIC indicated model with log transformed time (visit) provided the best model fit for PEP use, and linear time (visit) for PEP adherence.

Supplemental Table 7a: Regression Results for PEP Prescription in Past 4 Months by Arm & Time

**Variable**	**Odds Ratio Estimate**	**95% Posterior Interval for OR**
Intercept	0.03	0.02-0.04*
Coaching Arm	0.91	0.50-1.63
Peer Support Arm	0.68	0.38-1.24
Peer Support+Coaching Arm	0.71	0.38-1.24
Visit	0.42	0.35-0.51*
Visit × Coaching Arm	0.98	0.86-1.11
Visit × Peer Support Arm	1.01	0.89-1.15
Visit × Peer Support+Coaching Arm	1.03	0.89-1.15
log(Visit + 1)	28.44	15.63-52.18*

Supplemental Table 7b: Regression Results for PEP Adherence (took all doses) in past 4 months by Arm & Time

**Variable**	**Odds Ratio Estimate**	**95% Posterior Interval for OR**
Intercept	0.00	0.00-0.00*
Coaching Arm	1.46	0.37-5.70
Peer Support Arm	0.30	0.06-1.27
Peer Support+Coaching Arm	1.51	0.37-5.83
Visit	1.16	1.01-1.34*
Visit × Coaching Arm	0.94	0.74-1.17
Visit × Peer Support Arm	1.25	0.98-1.60
Visit × Peer Support+Coaching Arm	1.12	0.89-1.40

Supplemental Table 8a: Observed Percentages of Current PrEP Users at Each Study Assessment by Study Arm/Group for Analytic Sample (n=895) Corresponding to Main Text Figure 2 and Denominators in Figure 1.

Arm/Group	Baseline	4 Months	8 Months	12 Months	16 Months	20 Months	24 Months
AMMI	10.93%	15.27%	13.53%	12.70%	13.48%	14.35%	14.68%
AMMI + Coach	13.33%	17.18%	16.46%	12.88%	13.70%	16.78%	17.04%
AMMI + PS	11.27%	17.74%	16.18%	14.37%	15.58%	16.08%	16.11%
AMMI + PS + Coach	10.00%	17.79%	21.02%	23.36%	21.21%	22.58%	18.97%
Total sample	11.35%	16.77%	16.32%	15.20%	15.56%	16.96%	16.34%

Supplemental Table 8b: Estimated Percentages of Current PrEP Users at Each Study Assessment for Analytic Sample (n=895) by Study Arm/Group Corresponding to Figure 3 and Predicted Probabilities from GLMM Results in Table 2 in Main Text that Adjust for Missing Study Visit Data, Enrolment Date, and COVID-19 Onset

Arm/Group	Baseline	4 Months	8 Months	12 Months	16 Months	20 Months	24 Months
AMMI	11.35%	13.68%	13.87%	12.86%	11.91%	12.06%	14.50%
AMMI + Coach	13.75%	15.72%	15.39%	14.10%	12.88%	12.94%	15.50%
AMMI + PS	11.73%	15.38%	16.33%	15.42%	14.05%	13.39%	14.74%
AMMI + PS + Coach	10.48%	16.90%	20.37%	20.98%	20.11%	19.11%	19.74%
